# Surveillance and Epidemiology of Drug Resistant Infections Consortium (SEDRIC): Supporting the transition from strategy to action

**DOI:** 10.12688/wellcomeopenres.14586.2

**Published:** 2018-06-27

**Authors:** Keiji Fukuda, Direk Limmathurotsakul, Iruka N. Okeke, Nandini Shetty, Rogier van Doorn, Nicholas A. Feasey, Francesca Chiara, Ghada Zoubiane, Tim Jinks, Julian Parkhill, Jean Patel, Stuart W.J. Reid, Alison H. Holmes, Sharon J. Peacock, Surveillance and Epidemiology of Drug Resistant Infections Consortium (SEDRIC)

**Affiliations:** 1 School of Public Health, Li Ka Shing Faculty of Medicine, The University of Hong Kong, Pokfulum, Hong Kong; 2 Mahidol-Oxford Tropical Medicine Research Unit, Faculty of Tropical Medicine, Mahidol University, Bangkok, 10400, Thailand; 3 Department of Pharmaceutical Microbiology, Faculty of Pharmacy, University of Ibadan, Ibadan, Nigeria; 4 National Infection Service, Public Health England, London, NW9 5EQ, UK; 5 Oxford University Clinical Research Unit, National Hospital for Tropical Diseases, Hanoi, Vietnam; 6 The Malawi Liverpool Wellcome Trust Clinical Research Programme, Blantyre, Malawi; 7 Wellcome Trust, London, NW1 2BE, UK; 8 Wellcome Sanger Institute, Hinxton, CB10 1SA, UK; 9 Centers for Disease Control and Prevention, Atlanta, GA, 30333, USA; 10 Royal Veterinary College, Hatfield, AL9 7TA, UK; 11 Imperial College London, London, W12 0HS, UK; 12 London School of Hygiene and Tropical Medicine, London, WC1E 7HT, UK

**Keywords:** Surveillance, epidemiology, drug resistant infections, antimicrobial, SEDRIC

## Abstract

In recognition of the central importance of surveillance and epidemiology in the control of antimicrobial resistance and the need to strengthen surveillance at all levels, Wellcome has brought together a new international expert group SEDRIC (Surveillance and Epidemiology of Drug Resistant Infections Consortium). SEDRIC aims to advance and transform the ways of tracking, sharing and analysing rates of infection and antimicrobial resistance, burden of disease, information on antimicrobial use, opportunities for preventative measures such as vaccines, and contamination of the environment. SEDRIC aims to strengthen the availability of information needed to monitor and track risks, including an evaluation of access to, and utility of data generated by pharma and research activities, and will support the translation of surveillance data into interventions, changes in policy and more effective practices. Ways of working will include the provision of independent scientific analysis, advocacy and expert advice to groups, such as the Wellcome Drug Resistant Infection Priority Programme. A priority for SEDRIC’s first Working Group is to review mechanisms to strengthen the generation, collection, collation and dissemination of high quality data, together with finding more effective and creative uses of existing data and proxy measures, and linking such approaches to existing in-country capabilities. SEDRIC will also promote the translation of technological innovations into public health solutions.

## Introduction

The discovery, production and global use of antimicrobial drugs represents one of the most important achievements in medical history, and has contributed significantly to a major increase in life expectancy. Antimicrobial drugs continue to safeguard millions of lives each year, and ensuring access to quality medicines is key to continued gains in global health and development. However, widespread use of antimicrobials in humans, livestock and agriculture for health and non-health purposes in the 70 years since their commercial production has accelerated the emergence of drug-resistant lineages for the majority of common human pathogens worldwide.

The emergence and spread of antimicrobial resistance in the face of their extensive antibiotic use was both inevitable and predictable for rapidly replicating organisms like bacteria. This is based on the knowledge that bacteria develop antimicrobial agents to compete with other microorganisms in the natural environment, and therefore produce and use various resistance mechanisms to defend themselves. Genes encoding bacterial resistance to β-lactam, tetracycline and glycopeptide drugs have been found in ancient DNA from 30,000-year-old Beringian permafrost sediments ^1^. Humans who have never been treated with antimicrobial drugs are also colonised with bacteria that carry resistance genes, as shown by a study that detected a total of 28 different functional resistance genes in the microbiome of uncontacted Amerindians ^2^.

A major response to rising rates of bacterial resistance has been to develop new drug classes. Although a successful and necessary short-term strategy, this approach has inevitably selected new resistance patterns. Antimicrobial drug discovery itself has faltered as the string of easy wins for developing new drugs was exhausted and many pharmaceutical companies withdrew from their discovery ^3^. The combination of rising rates of bacterial resistance to commonly used antimicrobial drugs, their continued widespread and uncontrolled use and the lack of new therapeutic agents represents the perfect storm. Over the last 5 or so years there has been an increasing realisation of the scale of the global societal threat posed to human and animal health, agriculture, food and economic development. One of the pillars for sustainably reducing antimicrobial resistance worldwide is the need to measure and monitor resistance using appropriate surveillance systems.

A new and broader social and political awareness is now gathering pace. This has been reflected in the G20 summit of 2016 where leaders acknowledged the gravity of the threat and the need to stimulate new drug discovery and pursue international discussions; the launch of a global action plan to tackle antimicrobial resistance at the World Health Assembly in 2015; and the United Nations High Level Meeting on antimicrobial resistance and a General Assembly declaration in 2016 endorsing the plan and committing to take action. Aligned with this, the World Health Organization (WHO), Food and Agriculture Organization (FAO) and World Organization for Animal Health (OIE) have encouraged and supported countries to develop national action plans. The WHO has also developed a Global Antimicrobial Resistance Surveillance System (GLASS) to collect and report data on antimicrobial resistance rates aggregated at the national level and to develop international standards for surveillance to support stepwise participation by Member States over time ^4^.

At the same time, a firm long-term commitment to address antimicrobial resistance cannot be considered assured until this high level political strategy is translated into action across multiple sectors. The problem of antimicrobial resistance is acknowledged as being immensely complex. Nonetheless, clarity is being increasingly provided by numerous organizations, and several recent reviews ^5– 7^. Common themes include the need for a stronger discovery pipeline for new antimicrobial drugs and alternatives such as vaccines; the development of new point-of-care diagnostics; increased stewardship through the appropriate therapeutic use of antimicrobial drugs in humans and veterinary medicine; and the prevention of infections. Surveillance and epidemiology are widely recognised as critical cross-cutting foundational activities, and include mapping patterns of infection (including non-resistant infection) and the rates of drug resistant infections over time for a range of bug-drug combinations; estimation of the global economic, health and agricultural burden from drug resistant infections; mapping of the quality and quantity of antimicrobial drugs used in humans, animals and agriculture; the use and opportunity for better infection prevention and control, including vaccine coverage for the prevention of infectious diseases; and reduced contamination of the environment through better disposal practices and systems. The ideal surveillance system would integrate, or at the very least harmonize monitoring across the totality of the ecosystem – hosts, environment and putative routes of dissemination. Although significant efforts have been made to develop global antimicrobial resistance-related surveillance systems, current health, agriculture, veterinary and environmental approaches are fragmented and poorly coordinated, which limits their value. For harmonized surveillance to occur across sectors and countries, leadership by major international organizations and agreement across sectors to address and find ways to minimize differences among methodologies will be needed.

## SEDRIC

In recognition of the central importance of surveillance and epidemiology in the control of antimicrobial resistance and the need to strengthen and harmonize surveillance from local to global levels and across multiple sectors, especially health, agriculture and the environment, Wellcome has brought together a new international expert group termed SEDRIC (Surveillance and Epidemiology of Drug Resistant Infections Consortium) ^8^. SEDRIC aims to advance and where needed transform the ways that the global community are able to track, share and analyse information on antimicrobial use, together with rates of antimicrobial resistance and burden of disease and costs and, importantly, contamination of the environment. Its goal is to strengthen the availability of information needed to monitor and track risks, and to support the translation of surveillance data into interventions, changes in policy and more effective practices. Crucially, SEDRIC will have a particular focus on defining gaps in data and knowledge and barriers to the delivery of global surveillance functions, and on finding solutions to support existing systems such the WHO GLASS. Further details describing SEDRIC are summarised in Table 1.

**Table 1.** SEDRIC explained.

What is SEDRIC?	An international consortium of experts in the surveillance and epidemiology of drug resistant infections, and pathogens and infectious diseases more generally. A Board provides oversight and there is an inclusive membership of individual and institutional members.
Why was it created?	In recognition of the central importance of surveillance and epidemiology in the global effort to reduce drug resistant infections, and the need to strengthen surveillance at all levels from local to global and across multiple sectors including health, agriculture and the environment.
What is its purpose?	To strengthen the availability of information needed to monitor and track risks, and to support the translation of surveillance data into interventions, changes in policy and more effective practices.
How will it work?	SEDRIC will inform, influence and facilitate. Activities include a review series to highlight key challenges and actions to tackle this; working groups to undertake more detailed analyses of gaps, barriers and solutions; and an annual event for SEDRIC members to highlight activities, output and on-going priorities.
Excluded activities	SEDRIC is not a source of grant funding, and does not manage any aspect of research proposals.
Who can become an individual member?	People with expertise in any aspect of the surveillance and epidemiology of drug resistant infections or infectious disease more generally who will actively contribute to SEDRIC, including (but not limited to) the development and writing of reviews and opinion pieces, working groups, and commissioned work.
Contact details and webpage	sedric@wellcome.ac.uk https://wellcome.ac.uk/what-we-do/our-work/surveillance-and-epidemiology-drug-resistant-infections-consortium

The SEDRIC Board, which oversees the Consortium, includes 11 experts with extensive experience in human and veterinary health, drug-resistant infections and infectious pathogens and diseases more generally, including human and veterinary health, infection control and antimicrobial stewardship, laboratory microbiology, epidemiology, genomics, modelling, implementation and delivery of surveillance, and policy making. This group will be complemented by a wider network of members who are willing to contribute to the activities undertaken by SEDRIC, including the development and writing of reviews and opinion pieces, working groups, and commissioned work.

At the first SEDRIC Board meeting in January 2018, gaps and barriers to effective surveillance were identified and prioritised. The single most important short-term problem identified by these discussions was related to the need to intensify the generation, collection, collation and dissemination of high quality epidemiological data, together with the need for more creativity in the use of existing data and proxy measures, and linking to existing in-country capabilities. The collection of data across multiple sectors in a way that is useful, complementary and harmonized is a formidable challenge. Related issues include consideration of the optimal amount and frequency of data collection; standardised definitions and harmonised data collection methodology to permit comparability of findings; and the strengths and weaknesses of existing data collection tools, including their capacity for generalisability across a range of settings and levels of infrastructure. For example, an issue is how to relate patient outcome from bacterial infection data. A SEDRIC working group has been formed to further evaluate gaps and challenges relating to data and report on its findings and recommendations.

Allied to this data theme is consideration of how to identify, access and use available data from multiple sources. For example, large volumes of bacterial susceptibility data to numerous antimicrobial drugs are generated by pharmaceutical companies to fulfil regulatory requirements. Extensive information is also generated through research. Many of these data are likely to be of high quality but are generally not being utilized for surveillance purposes. In low and middle-income countries (LMIC), available quality-assured data originates mainly from individual research projects in well-funded academic institutions or the private sector ^9^. Where other sources of surveillance information are currently limited, use of data from such sources could be of considerable benefit. Whilst it is widely acknowledged that developing a reliance on data generated by research or from the private sector has its drawbacks in terms of generalisability and sustainability, ignoring such data is also unwise. Such data could provide significant information in the interim to countries that are actively working towards capacity building and training in critical areas such as microbiology. A SEDRIC-led paper to be published in the coming months will discuss this in more detail.

SEDRIC will also promote the translation of technological innovations into public health solutions in alignment with Wellcome’s goal to support and facilitate the rapid advancement of discoveries into improving health globally. For example, innovations could significantly enhance surveillance of drug resistant infections. This includes the development and widespread use of new laboratory instruments that are robust, simple to use and provide accurate bacterial identification and testing of their susceptibility; the development of data capture tools using easier and enhanced connectivity including innovative data interfaces that are designed to reflect end-user inputs and experience; and the application of Artificial Intelligence methodologies. Such approaches can also be applied to capturing trends in antimicrobial use and overall patterns of infectious diseases. SEDRIC is particularly interested in ‘leapfrog’ technologies, especially those that are suitable and accessible for LMIC settings. SEDRIC will specifically consider the role of pathogen genome sequencing as an integral component of surveillance and epidemiology. Such data can contribute to a more accurate and complete picture of the global emergence and spread of pathogens, identify the genetic basis for phenotypic resistance, and track novel resistance mechanisms as they emerge. Numerous research groups and some national surveillance programmes are actively engaged in sequencing bacterial collections from around the world, but efforts are largely fragmented and data are generally not combined, limiting their use. Most broadly, SEDRIC will support and advocate for the translation of data into interventions and policy changes. Such outcomes are critical for “pulling” along transformation, change and increasing willingness to coordinate efforts. It is crucial that data from GLASS and multiple surveillance sources are critically analysed and translated into actions at both country and global levels at a pace that moves faster than the development of antimicrobial resistance and brings benefit to health.

## Disclaimer

The views expressed in this article are those of the author(s). Publication in Wellcome Open Research does not imply endorsement by Wellcome. The findings and conclusions in this report are those of the author(s) and do not necessarily represent the official position of the Centers for Disease Control and Prevention.

## Data availability

No data are associated with this article.
