# Supplementary material for: Surveillance and Epidemiology of Drug Resistant Infections Consortium (SEDRIC): Supporting the transition from strategy to action
Source: Wellcome Open Res. 2018 Jun 27;3:59. Originally published 2018 May 16. [Version 2] doi: 10.12688/wellcomeopenres.14586.2 (PMC5989141; doi:10.12688/wellcomeopenres.14586.2)
Supplement: Peer review history

# Peer review: Referee response for version 2

**DOI:** 10.21956/wellcomeopenres.15992.r33424

**Published:** 2018-09-03

**Authors:** Carmem L. Pessoa-Silva

**Affiliations:** 1 AMR Secretariat, World Health Organization (WHO), Geneva, Switzerland

Please see below the explanation for each of the points appraised.

**Is the rationale for the Open Letter provided in sufficient detail?**

 Yes. The paper announces a new initiative and clearly states that it represents the views of a Wellcome-funded group.

**Does the article adequately reference differing views and opinions?**

 Yes.

**Are all factual statements correct, and are statements and arguments made adequately supported by citations?**

 Partly. Whilst the use of existing data generated by pharmaceutical companies does require serious consideration, the statement of the former data being “high quality” is an assumption and may need to be supported by citations.

**Is the Open Letter written in accessible language?**

 Yes

**Where applicable, are recommendations and next steps explained clearly for others to follow?**

 Yes. Most intentions and next steps are clearly explained, but some could benefit from further clarification: Although it seems to be clear form the paper that the initiative will focus on bacterial infections and antibacterial resistance, the inconsistent use of the terms “drug resistance”, “antimicrobial resistance”, “antibiotic resistance” may suggest a broader scope which needs to be better defined. 3rd paragraph under ‘SEDRIC’ and Abstract. The stated priority of linking data to “existing in-country networking infrastructure” is rather vague. The authors could support with examples or citations for better understanding by the audience.

I have read this submission. I believe that I have an appropriate level of expertise to confirm that it is of an acceptable scientific standard.

# Peer review: Referee response for version 1

**DOI:** 10.21956/wellcomeopenres.15881.r33159

**Published:** 2018-06-01

**Authors:** Carmem L. Pessoa-Silva

**Affiliations:** 1 AMR Secretariat, World Health Organization (WHO), Geneva, Switzerland

Please see below the explanation for each of the points appraised.

**Is the rationale for the Open Letter provided in sufficient detail?**

 Yes. The paper announces a new initiative and clearly states that it represents the views of a Wellcome-funded group.

**Does the article adequately reference differing views and opinions?**

 The article provides good references. It could benefit of expanding on the differences in surveillance approaches.

 While substantial portion of the introduction is devoted to description of the evolution of antimicrobial resistance and its challenges and increasing political awareness, the methodological gaps in most existing surveillance approaches and their implications could have been more clearly described to justify and better explain the proposed innovations.

 The need for surveillance is indeed “widely recognised as critical cross-cutting activity”, but the importance of and emphasis on well-designed epidemiological methods is still lacking and most existing surveillance initiatives are producing predominantly microbiological data (a good example is the reference 9). Currently, most surveillance initiatives provide information only on proportion of resistant bacteria among tested isolates, and frequently without any patient information nor elimination of duplicated results. The recently launched WHO Global AMR Surveillance System (GLASS) also faces this challenge. Although GLASS proposes a methodology to include information on the sampled population, yet in the first GLASS report issued in January 2018, only 5 out of the 22 countries reporting AMR rates could provide this type of information. So, the SEDRIC initiative could be an excellent opportunity to promote sound epidemiological methods to drive AMR surveillance.

**Are all factual statements correct, and are statements and arguments made adequately supported by citations?**

 Whilst the use of existing data generated by pharmaceutical companies does require serious consideration, the statement of the former data being “high quality” is an assumption and may need to be supported by citations.

**Is the Open Letter written in accessible language?**

 Yes

**Where applicable, are recommendations and next steps explained clearly for others to follow?**

 Most intentions and next steps are clearly explained, but some could benefit from further clarification:

- Although it seems to be clear form the paper that the initiative will focus on bacterial infections and antibacterial resistance, the inconsistent use of the terms “drug resistance”, “antimicrobial resistance”, “antibiotic resistance” may suggest a broader scope which needs to be better defined.
- 3 ^rd^ paragraph under ‘SEDRIC’ and Abstract. The stated priority of linking data to “existing in-country networking infrastructure” is rather vague. The authors could support with examples or citations for better understanding by the audience.

I have read this submission. I believe that I have an appropriate level of expertise to confirm that it is of an acceptable scientific standard, however I have significant reservations, as outlined above.

# Response

**Published:** 2018-06-22

**Authors:** Sharon Peacock

**Affiliations:** London School of Hygiene & Tropical Medicine, UK

We thank the referee for their thoughtful comments. We hope that the revisions made are sufficient and satisfactory. The main changes made are described below.

**Does the article adequately reference differing views and opinions?**

 The article provides good references. It could benefit of expanding on the differences in surveillance approaches.

*Whilst this paper is not an analysis of methodological issues per se but a statement of where SEDRIC plans to focus, we have highlighted methodological concerns in an edited draft of the paper. This includes the following (last paragraph of the introduction):*

*The ideal surveillance system would integrate, or at the very least harmonize monitoring across the totality of the ecosystem – hosts, environment and putative routes of dissemination. Although significant efforts have been made to develop global antimicrobial resistance-related surveillance systems, current health, agriculture, veterinary and environmental approaches are fragmented and poorly coordinated, which limits their value. For harmonized surveillance to occur across sectors and countries, leadership by major international organizations and agreement across sectors to address and find ways to minimize differences among methodologies will be needed.*

  While substantial portion of the introduction is devoted to description of the evolution of antimicrobial resistance and its challenges and increasing political awareness, the methodological gaps in most existing surveillance approaches and their implications could have been more clearly described to justify and better explain the proposed innovations.

*The edited draft provides further justification, as described above.*

 The need for surveillance is indeed “widely recognised as critical cross-cutting activity”, but the importance of and emphasis on well-designed epidemiological methods is still lacking and most existing surveillance initiatives are producing predominantly microbiological data (a good example is the reference 9). Currently, most surveillance initiatives provide information only on proportion of resistant bacteria among tested isolates, and frequently without any patient information nor elimination of duplicated results. The recently launched WHO Global AMR Surveillance System (GLASS) also faces this challenge. Although GLASS proposes a methodology to include information on the sampled population, yet in the first GLASS report issued in January 2018, only 5 out of the 22 countries reporting AMR rates could provide this type of information. So, the SEDRIC initiative could be an excellent opportunity to promote sound epidemiological methods to drive AMR surveillance.

*We thank the reviewer for this comment.*

**Are all factual statements correct, and are statements and arguments made adequately supported by citations?**

 Whilst the use of existing data generated by pharmaceutical companies does require serious consideration, the statement of the former data being “high quality” is an assumption and may need to be supported by citations.

*This has been reworded to address the point made by removing reference to ‘high quality’.*

**Where applicable, are recommendations and next steps explained clearly for others to follow?**

 Most intentions and next steps are clearly explained, but some could benefit from further clarification:

 Although it seems to be clear form the paper that the initiative will focus on bacterial infections and antibacterial resistance, the inconsistent use of the terms “drug resistance”, “antimicrobial resistance”, “antibiotic resistance” may suggest a broader scope which needs to be better defined.

*We have rationalised the use of these terms, removing reference to antibiotic resistance and referring to antimicrobial resistance throughout. We have continued to use the term drug-resistant infections to highlight the difference between an organism that is resistant to antimicrobial drugs, and an infection caused by such an organism.*

 3 ^rd^ paragraph under ‘SEDRIC’ and Abstract. The stated priority of linking data to “existing in-country networking infrastructure” is rather vague. The authors could support with examples or citations for better understanding by the audience.

*We agree that this was unclear and have redrafted for clarity, changing this to ‘existing in-country capabilities’.*

# Peer review: Referee response for version 1

**DOI:** 10.21956/wellcomeopenres.15881.r33162

**Published:** 2018-06-01

**Authors:** Kathryn E. Holt

**Affiliations:** 1 Department of Biochemistry and Molecular Biology, Bio21 Molecular Science and Biotechnology Institute (Bio21 Institute), University of Melbourne, Parkville, Vic, Australia

This open letter introduces SEDRIC, the Surveillance and Epidemiology of Drug Resistant Infections Consortium. The rationale, goals and activities of the consortium are clearly laid out in accessible language, along with details of how interested parties can join the consortium and why they may wish to do so.

 The background information provided is accurate and adequately referenced, and clearly sets the scene for the creation of the consortium. The letter clearly articulates how the consortium plans to interact with other players in this space, including governments, WHO, and the funding arm of Wellcome.

 I have no criticisms and believe that no further changes are required to make the article scientifically sound.

I have read this submission. I believe that I have an appropriate level of expertise to confirm that it is of an acceptable scientific standard.

# Response

**Published:** 2018-06-21

**Authors:** Sharon Peacock

**Affiliations:** London School of Hygiene & Tropical Medicine, UK

We thank the reviewer for taking the time to read and comment on our article.

# Peer review: Referee response for version 1

**DOI:** 10.21956/wellcomeopenres.15881.r33161

**Published:** 2018-05-25

**Authors:** Samuel Kariuki, Robert Onsare

**Affiliations:** 1 Centre of Microbiology Research, Kenya Medical Research Institute, Nairobi, Kenya; 2 Kenya Medical Research Institute, Nairobi, Kenya

This is a report that sets out to spell the terms of engagement for a newly launched initiative consisting of an international expert group named SEDRIC (Surveillance and Epidemiology of Drug Resistant Infections Consortium). SEDRIC aims to advance and transform the ways of tracking, sharing and analysing rates of infection and drug resistance, burden of disease, information on antibiotic use, opportunities for preventative measures such as vaccines, and contamination of the environment. It will be imperative that the group take cognizance of the numerous other initiatives globally that also seek to determine AMR burden in infectious disease epidemiology globally, what can make a difference in reversing the global trends, and support for efforts to develop new antimicrobials. It will be critical that a multidisciplinary task force/forum be set up through which all the AMR interest groups can share data in an effort to harmonize activities and avoid duplication of effort.

We have read this submission. We believe that we have an appropriate level of expertise to confirm that it is of an acceptable scientific standard.

# Response

**Published:** 2018-06-21

**Authors:** Sharon Peacock

**Affiliations:** London School of Hygiene & Tropical Medicine, UK

We thank the reviewer for his comments. We fully agree that we should take cognizance of the numerous other initiatives globally that also seek to determine AMR burden in infectious disease epidemiology globally, what can make a difference in reversing the global trends, and support for efforts to develop new antimicrobials. SEDRIC will seek to facilitate and support existing efforts and avoid duplication, as well as promoting the translation of technological innovations into public health solutions.
